# Isolation and Characterization of Cultivable Microbes from the Gut of *Zophobas atratus* (Coleoptera: Tenebrionidae) Larvae Reared on Two Types of Artificial Diets

**DOI:** 10.3390/biology14070824

**Published:** 2025-07-07

**Authors:** Vladislava Baklanova, Alexander Kuprin, Ivan Baklanov, Vadim Kumeiko

**Affiliations:** 1 Federal Scientific Center of the East Asia Terrestrial Biodiversity, Far East Branch of the Russian Academy of Sciences, Vladivostok 690022, Russia; 2 School of Medicine and Life Sciences, Far Eastern Federal University, Vladivostok 690922, Russia; 3 A.V. Zhirmunsky National Scientific Center of Marine Biology, Far East Branch of the Russian Academy of Sciences, Vladivostok 690041, Russia

**Keywords:** diet, development, culturable microbes, microbial isolation, Tenebrionidae, Coleoptera, *Zophobas atratus* (Fabricius, 1775)

## Abstract

Gut microbes are important for saproxylophagous insects, but little is known about the specific types of microbes that we can grow in the lab and how their diet affects them. We characterized aerobic culturable microbes from the superworm *Zophobas atratus* larvae reared on a standard diet (SD) and a fungal-based diet (FD) using the selective plating and 16S rRNA sequencing of isolates. Five functional groups were cultured: amino acid autotrophs, enterobacteria, yeasts, cellulolytic bacteria, and molds. A quantitative assessment revealed distinct diet-dependent patterns: SD-fed larvae showed the dominance of enterobacteria and amino acid autotrophs, while FD-fed larvae exhibited a higher abundance of enterobacteria and yeasts. Mold populations remained minimal under both diets. A phylogenetic analysis of bacterial isolates showed four core bacterial phyla (*Pseudomonadota*, *Actinobacteria*, *Bacillota*, and *Bacteroidota*) with diet-sensitive genus-level variations. *Pseudomonadota* dominated both diets, but certain genera were associated with different diets: *Micrococcus* and *Brucella* in the SD versus *Citrobacter* and *Pseudomonas* in the FD. Shared genera (*Klebsiella*, *Enterobacter*, and *Bacillus*) may represent a core culturable community. These findings demonstrate the influence of diet on culturable gut microbes while highlighting the need for complementary molecular approaches to study unculturable taxa. The isolated strains provide resources for investigating microbial functions in insect nutrition.

## Simple Summary

Microbes in the gut of insects contribute to the metabolism of nutrients and adaptation to the diet. In this study, we characterized how standard and fungal-based diets affect aerobic culturable microorganisms isolated from the superworm *Zophobas atratus* larvae. Using culture-dependent methods, we identified key microbial groups, including amino acid autotrophs, enterobacteria, cellulolytic bacteria, yeasts, and molds, showing diet-dependent differences in their culturability. The 16S rRNA gene sequencing of bacterial isolates revealed four phyla (*Pseudomonadota*, *Actinobacteria*, *Bacillota*, and *Bacteroidota*) with diet-associated variations at the genus level. These results demonstrate the influence of diet on culturable gut microbes, providing isolated strains for future functional studies while recognizing that this represents only part of the microbial community.

## 1. Introduction

Insects represent the most diverse and abundant group of animals, inhabiting nearly all ecological niches [1]. The most critical factor contributing to the biological success of this diverse group is their association with microbes [2]. Symbiotic relationships between insects and microbes have influenced insect evolution [3,4,5], shaping dietary adaptations [6] and broad ecological specialization [7]. For example, symbiotic microbes allow their insect hosts access to otherwise indigestible nutrients, and they support vital physiological functions, including modulation of the immune system; host behavior; reproductive success; defense against predators, parasites, and pathogens; and interspecific and intraspecific communication [8,9,10,11,12,13]. Mutualism is particularly pronounced in wood-inhabiting insects, where the nature of such symbiotic relationships depends on the host’s dietary specialization [14,15]. Microbial consortia—comprising both bacteria and fungi—play complementary roles in nutrient acquisition in wood-feeding insects [16]. While bacteria dominate gut communities, fungi contribute to the initial breakdown of recalcitrant lignocellulose through extracellular enzymatic “predigestion”, releasing metabolites that bacteria subsequently metabolize [17]. This cross-kingdom synergy enhances nutrient accessibility for the host [18,19].

In a previous study, we developed an artificial fungal wood diet to rear the rare and endangered longhorn beetle *Callipogon relictus* Semenov, 1899 (Coleoptera: Cerambycidae), whose larvae develop in highly decomposed wood [20]. Such wood decomposition involves not only bacteria but also xylotrophic fungi, which perform critical functions: mycogenic xylolysis, vitamin production, and the synthesis of bioactive secondary metabolites (e.g., sterols and phenolic compounds) that facilitate the development and adaptation of xylophagous insects [21,22]. We also examined the effects of temperature and humidity on the beetle’s developmental rate under laboratory conditions, demonstrating several advantages of this diet for the preservation of rare saproxylic beetles [23,24]. Since *C. relictus* is protected under international agreements in the Republic of Korea and the Red Data Book of Russia, with wild larval collection prohibited [25,26], subsequent studies on xylophagous insect adaptations to this fungal wood diet were conducted using an alternative model organism [27]. We selected *Zophobas atratus* (Fabricius, 1775) (Coleoptera: Tenebrionidae) as a model species because darkling beetle larvae share trophic preferences with longhorn beetles in natural environments, frequently consuming lignin-rich substrates colonized by wood-decay fungi [28].

The darkling beetle *Z. atratus* is a widely distributed and ecologically significant insect species [29]. Its larvae—saprophagous organisms commonly known as “superworms”—exhibit a broad dietary tolerance, typically feeding on decaying plant matter, including fungal-colonized lignin-rich substrates [30]. *Z. atratus* has increasingly been utilized as a model organism in studies of insect physiology, nutrition, and immunity due to its ease of laboratory rearing, rapid life cycle, and ability to thrive on diverse diets [31,32,33,34]. Furthermore, *Z. atratus* larvae have garnered attention for their bioremediation and waste management potential, demonstrating the capacity to degrade various pollutants, including polystyrene [35,36,37]. Despite these findings, the influence of different diets—particularly those involving fungal-mediated lignin decomposition—on the gut microbiota composition and its functional consequences in *Z. atratus* remains insufficiently studied. Understanding insect gut microbial communities is crucial for elucidating the mechanisms of trophic specialization, environmental adaptation strategies, mutualistic relationships, and biotechnology applications [14,38].

While molecular approaches have advanced our understanding of insect gut communities [39], microbial culture approaches remain essential for functional characterization [40]. We previously showed that the larval diet affects the *Z. atratus* larval development rate, molting dynamics, and hemolymph antibacterial activity, thereby enhancing immune responses [41], but the culturable microbial fraction remains unexplored. Most studies focus on community profiling rather than isolate collection, despite the need for cultured strains to investigate microbial functions [40,42,43].

The objective of the present study is to examine the impact of distinct dietary regimens—including fungal-supplemented lignin substrates—on culturable aerobic and facultatively anaerobic microbes in *Z. atratus* larvae. Using established culture methods, we quantify major physiological groups across diets, characterize bacterial isolates phylogenetically, and identify diet-associated patterns in cultured taxa.

This approach provides isolates for functional studies while acknowledging that culture-based methods capture only part of gut microbial diversity [39]. The isolates obtained will facilitate future investigations into microbial roles in insect nutrition and adaptation to lignocellulosic diets.

## 2. Materials and Methods

### 2.1. Zophobas atratus Origin and Rearing on Experimental Diets

The insects used in this study originated from a breeding colony established from ten pairs of adult beetles from the bioresource collection at the Federal Centre for Biodiversity of the Far Eastern Branch of the Russian Academy of Sciences in Vladivostok, Primorsky Krai, Russia. The beetles were maintained in 5 L plastic containers provisioned with a 5 cm base layer of Japanese elm (*Ulmus japonica* (Rosale: Ulmaceae)) sawdust, leaf litter to maintain optimal humidity levels, and large wooden branches serving the dual purpose of shelter and oviposition sites [20]. Following oviposition, eggs were carefully collected and transferred to specialized Ferplast Geo Large rearing chambers (Rome, Italy) containing one of the experimental diets (see Table 1). The eggs were then incubated in an MIR-154 incubator (Sanyo, Tokyo, Japan) under controlled laboratory conditions at 26–28 °C, 60–70% relative humidity, and a 12:12 h light–dark (L12:D12) photoperiod. To prevent desiccation, the substrate surface was misted with distilled water 2–3 times weekly.

The fungal-based diet (FD) was specifically designed to match the natural feeding preferences of tenebrionid beetles and their saproxylic larvae [29]. For its preparation, we used ground wood from *U. japonica* with a moisture content of 60–70%, which was sterilized in a GC-100-3 steam sterilizer (AO TZMOI, Moscow, Russia) at 121 °C for 2 h under 1 atmosphere of steam pressure. After cooling to room temperature, the wood chips were supplemented with *Pleurotus citrinopileatus* (Agaricales: Pleurotaceae) mycelium, feed-grade yeast, ascorbic acid, sucrose, agar, and distilled water using the proportions indicated in Table 1 [41]. The mixture was incubated in complete darkness in an MIR-154 cooled incubator at 25 °C and 70% relative humidity for approximately 20 days to allow for fungal mycelium development, following the protocol established for *C. relictus* [14]. As a control diet, we used autoclaved *U. japonica* sawdust without *P. citrinopileatus* mycelium. The standard diet (SD), conventionally used to rear edible insects, consisted of wheat flakes and bran that were microwave-treated at 100 °C with cyclic heating to ensure sterilization while preserving nutritional quality [44].

The larval rearing protocol spanned nearly two years (encompassing three generations, with 7-month life cycles each), ensuring sufficient biomass accumulation for comprehensive analyses. Experimental groups consisted of 10 first-instar larvae per 500 g of substrate, with three replicates established for each condition. However, first-instar larvae failed to survive on the control diet (showing no feeding activity and dying within 3–4 weeks), precluding its use in further analyses. For intestinal tissue sampling, a total of 60 twelfth-instar larvae were processed (10 individuals per technical replicate). All experimental procedures were conducted under standardized laboratory conditions.

### 2.2. Obtaining Intestinal Tract Tissues from Zophobas atratus Larvae

To obtain intestinal tissues, the larvae were placed individually in substrate-free Petri dishes and subjected to a 3-day fasting period to clear the gut contents, thereby eliminating transient bacterial populations. Subsequently, the larval surfaces were decontaminated by washing with 70% ethanol to remove external debris and surface microbiota. Subsequent rinsing with sterile physiological saline (0.9% NaCl solution) eliminated residual ethanol. Using a sterile scalpel and forceps, we performed intestinal dissection. Ten complete larval digestive tracts were homogenized, and the resulting suspension was vortexed for thorough mixing. The homogenate was allowed to settle for 10–15 min to facilitate particulate sedimentation. To reduce the microbial concentration, the suspension was processed through serial dilution techniques. Specifically, the original suspension was sequentially diluted tenfold (10^−1^, 10^−2^, 10^−3^, etc.) using sterile physiological saline (0.9% NaCl), resulting in an exponential decrease in the microbial density [45].

### 2.3. Analysis of Culturable Aerobic/Facultative Anaerobic Microbial Groups from Zophobas atratus Larval Gut

To identify physiological groups of microorganisms associated with the intestinal tract of *Z. atratus* larvae, we plated 100 μL aliquots from each serial dilution (including the undiluted sample) onto selective culture media. Microbial groups were differentiated based on their growth in specific media: amino acid autotrophs—fishmeal hydrolysate agar (FBIS SRCAMB, Obninsk, Russia), enterobacteria—Endo agar (FBIS SRCAMB, Obninsk, Russia), fungi—Sabouraud agar (FBIS SRCAMB, Obninsk, Russia), and cellulolytic bacteria—Hutchinson’s medium supplemented with filter paper as the sole carbon source (FBIS SRCAMB, Obninsk, Russia). The plates were incubated at 28 °C for 5 days in an ES-20/60 incubator (Biosan, Riga, Latvia). The combination of selected culture media enabled comprehensive isolation of the principal culturable functional groups present in the *Z. atratus* gut microbiota.

For each physiological group of microorganisms, we calculated the colony-forming units (CFU) per 1 g of larval intestine (CFU/g) using the following methodology: Colonies were counted on Petri dishes containing 30–300 CFU whenever possible to ensure measurement accuracy. A critical requirement was using only plates with well-isolated and clearly distinguishable colonies for counting. The microbial concentration in the original suspension (CFU/mL) was determined using the formula CFU/mL = colony count × dilution factor × 10. In this formula, colony count represents the number of colonies on a specific Petri dish; the dilution factor indicates the sample dilution degree (e.g., 10^3^ for a 10^−3^ dilution); and the multiplier 10 serves as a correction factor to convert to 1 mL, accounting for the inoculation volume (100 μL per plate). To convert the results to per gram of tissue, we used an additional formula, CFU/g = CFU/mL × 10, where the multiplier 10 reflects the initial sample dilution (1 g of intestinal tissue in 10 mL of physiological saline) [45,46].

### 2.4. Analysis of Cultured Aerobic/Facultative Anaerobic Bacterial Isolates from Zophobas atratus Larvae Gut

We isolated pure microbial strains exhibiting distinct morphotypes based on macro- and micro-rearing characteristics using mechanical separation on agar-solidified media from primary bacterial cultures. Culture purity was verified through a visual inspection of colony morphology and a microscopic examination. The homogeneity of the grown colonies was utilized as the criterion for determining purity. The cultural properties (i.e., growth characteristics on different media) of all isolated bacterial strains were documented, as were their tinctorial properties (i.e., staining characteristics) and morphological features (i.e., cell shape, size, and arrangement) [47].

For Sanger sequencing, genomic DNA was extracted from bacterial cultures using an NK-sorbent Base nucleic acid isolation kit (Litech, Moscow, Russia) for in vitro diagnostics, following the manufacturer’s protocol. The 16S rRNA gene fragment was amplified using a BioMaster HS-Taq PCR-Color (2×) kit (Biolabmix, Novosibirsk, Russia) and universal bacterial primer pairs: 27F (5′-AGAGTTTGATCATGGCTCAG-3′) with 1350R (5′-GACGGGCGGTGTGTACAAG-3′) and 27F (5′-AGAGTTTGATCATGGCTCAG-3′) with 1492R (5′-TACGGCTACCTTGTTACGA-3′) [48].

Amplification was performed using a T100 Thermal Cycler (Bio-Rad, Hercules, CA, USA) with the following cycling protocol: initial denaturation 4 min at 94 °C (1 cycle); 60 s at 94 °C, 60 s at 48 °C, 90 s at 72 °C (5 cycles); 60 s at 92 °C, 110 s at 50 °C, 90 s at 72 °C (10 cycles); 60 s at 92 °C, 60 s at 52 °C, 60 s at 72 °C (10 cycles); 60 s at 92 °C, 60 s at 54 °C, 110 s at 72 °C (10 cycles); final extension 10 min at 72 °C (1 cycle). The verification of PCR products was performed by gel electrophoresis in 1% agarose gel (at ~2 V/cm) containing ethidium bromide (2 μg/mL). The results were visualized using a transilluminator UView (Bio-Rad, USA). The purification of PCR products was achieved using a ExoSAP-IT Express kit (Thermo Fisher Scientific Inc., Waltham, MA, USA), a process that was undertaken to ensure the removal of residual reaction components.

The purified amplification products (16S rRNA fragments) were subjected to Sanger sequencing using an ABI 3500 Genetic Analyzer (Applied Biosystems, Waltham, MA, USA; the Instrumental Centre for Biotechnology and Gene Engineering, FSCEATB FEB RAS) with a BigDye Terminator v3.1 Cycle Sequencing Kit (Thermo Fisher Scientific Inc., Waltham, MA, USA), according to the manufacturer’s protocols.

The sequences were analyzed using the Staden Package software (version 1.4) [49]. Base calling was performed through a fluorescence trace data analysis (Phred-based) in pregap4 software. Chromatograms were visually inspected, sequences were assembled, and contigs were edited using gap4 software.

The taxonomic assignment of bacterial strains to specific genera was performed by searching for homologous/identical sequences in the GenBank database using the BLAST algorithm (http://www.ncbi.nlm.nih.gov/blast, accessed 1 January 2025). Sequence alignment was conducted with the Clustal W algorithm (ver. 1.81). Phylogenetic reconstruction was performed using the Neighbor-Joining method [50], with branch support assessed through a bootstrap analysis (500 replicates) [51,52]. All phylogenetic analyses were carried out using MEGA 11 software [53].

### 2.5. Statistical Analysis

Statistical analyses were performed using Statistica 10 (StatSoft, Round Rock, TX, USA) and GraphPad Prism 8 (GraphPad Holdings, Boston, MA, USA) software packages. The Shapiro–Wilk test was used to assess data normality and revealed significant deviation from a normal distribution (*p* < 0.05). Consequently, non-parametric statistical methods were employed for subsequent analyses. Quantitative differences between two independent groups (FD and SD samples) were evaluated using the Mann–Whitney U test. Differences were considered statistically significant at *p* < 0.05.

## 3. Results

**Figure 1:**
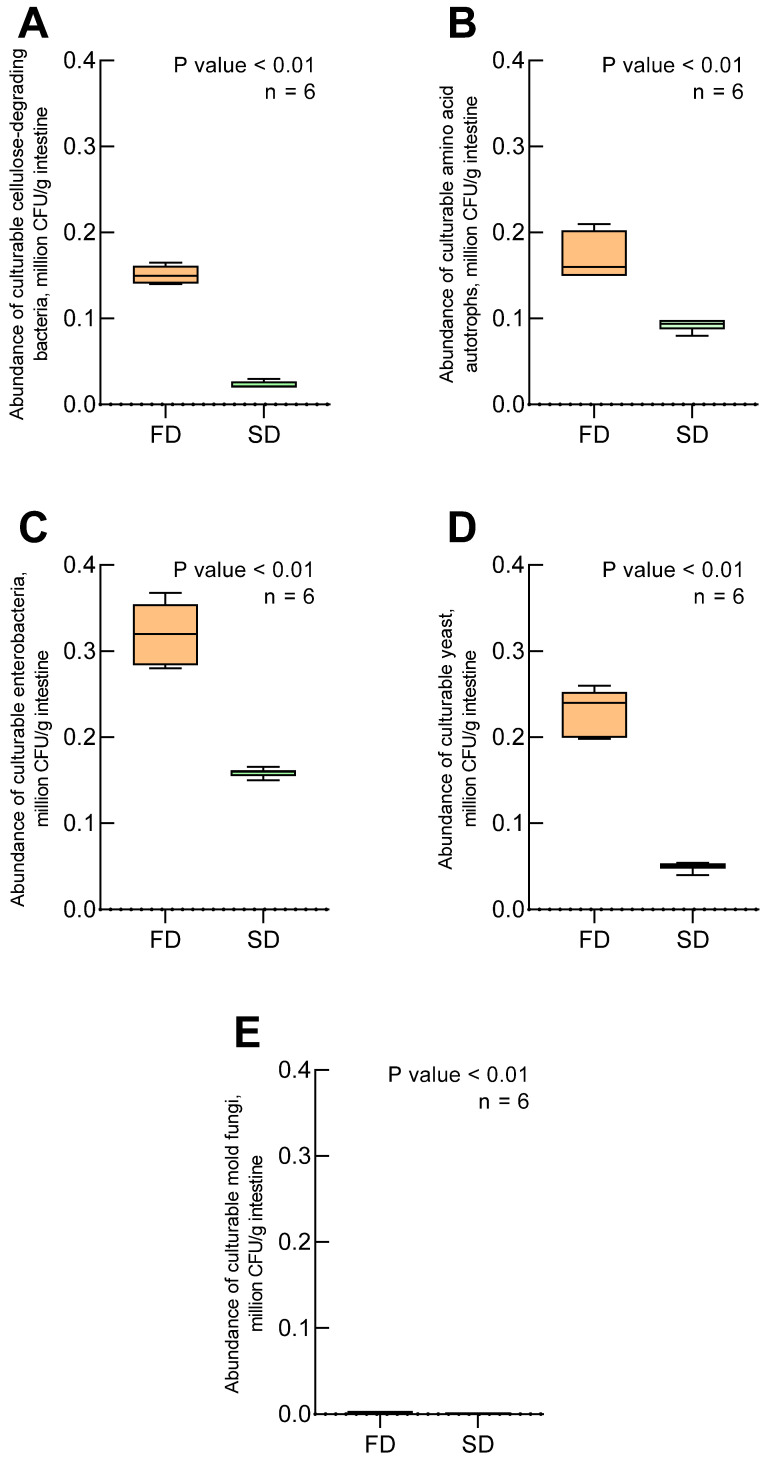
Abundance of culturable microbial groups isolated from *Zophobas atratus* larval gut on selective media: (**A**) cellulose-degrading bacteria, (**B**) amino acid autotrophs, (**C**) enterobacteria, (**D**) yeast, (**E**) mold fungi. SD—standard diet; FD—fungal-based diet; *n* = 6 independent replicates; *p* value < 0.01—statistical significance determined using the Mann–Whitney U test. Note: Data represent colony-forming units (CFU) on specific culture media and may not reflect in vivo abundances.

The intestinal tract of the larvae in both experimental groups was found to contain five distinct microbial groups: cellulose-degrading bacteria (Figure 1A), amino acid autotrophs (Figure 1B), enterobacteria (Figure 1C), yeasts (Figure 1D), and mold fungi (Figure 1E). All identified microorganisms were aerobes or facultative anaerobes, while obligate anaerobes were not investigated in this study. In the larvae reared on the SD, enterobacteria (74.33 × 10^3^ CFU/g) and amino acid autotrophs (441.33 × 10^3^ CFU/g) constituted the most abundant groups. In contrast, the larvae fed the FD showed a predominance of enterobacteria and yeasts (Figure 1). Molds represented the least abundant cultivable microbial group in both samples, with counts of 2.67 × 10^3^ CFU/g and 1.75 × 10^3^ CFU/g, though their species composition was not examined in this study.

A working collection of 29 bacterial isolates was established, comprising 10 isolates from the intestinal tract of the larvae reared on the SD and 19 isolates from the larvae fed the FD. All cultures were characterized as aerobes or facultative anaerobes, demonstrating robust growth on peptone agar within a temperature range of 25–37 °C, with optimal growth at pH 7.0. Notably, bacterial growth was not inhibited by coexisting microscopic fungi. The isolates exhibited characteristic cultural, tinctorial, and morphological properties consistent with their taxonomic identification.

**Figure 2:**
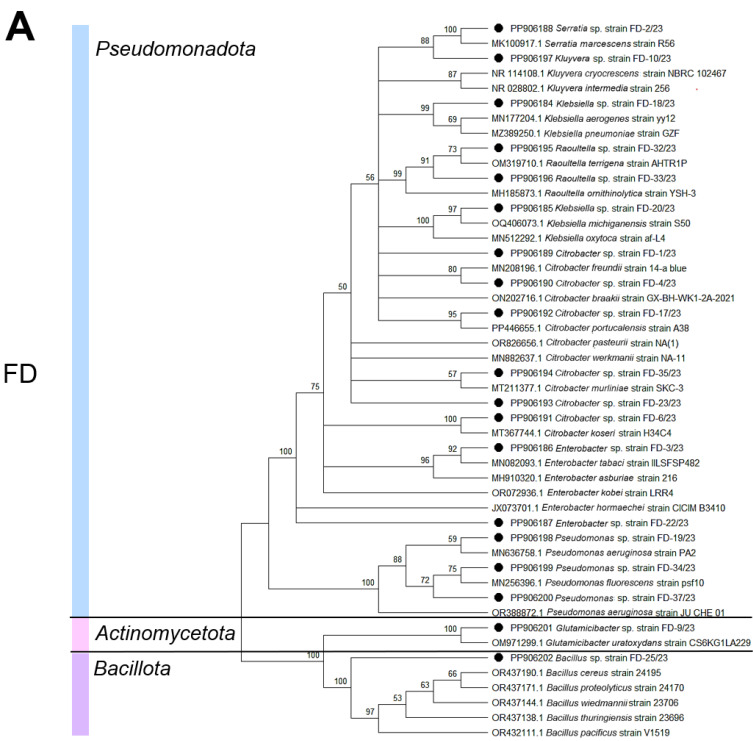

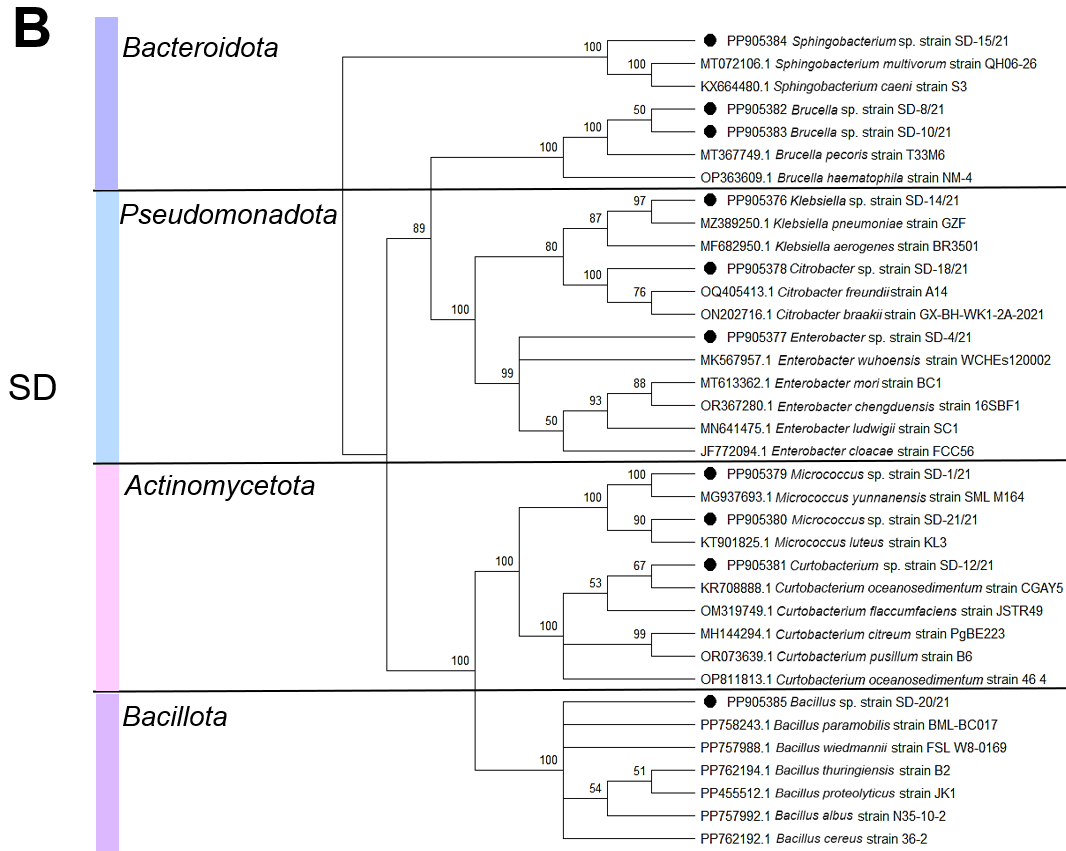
Diet-associated differences in cultured bacterial isolates from *Zophobas atratus* larvae. Phylogenetic trees based on 16S rRNA sequences of bacterial strains isolated from larvae reared on (**A**) fungal and (**B**) standard diets. Black circles indicate sequences obtained in this study (bootstrap values > 50% shown). Note: Analysis limited to culturable aerobic/facultative anaerobic bacteria.

**Figure 3:**
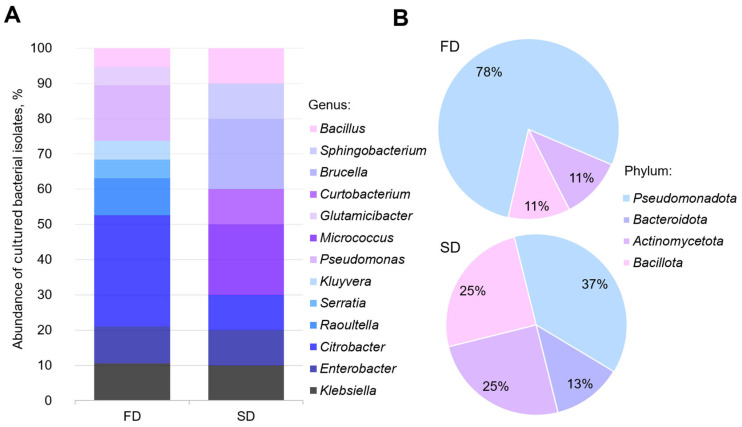
Diet-related patterns in cultured bacterial isolates from *Zophobas atratus* larvae: (**A**) genus-level distribution of isolates; (**B**) phylum-level distribution of isolates. Note: Charts reflect relative proportions of cultured isolates, not total microbial community composition.

We obtained and deposited the 16S rRNA gene sequences of the bacterial strains isolated from the *Z. atratus* larvae in the GenBank database under accession numbers PP905376-PP905385 and PP906184-PP906202 (Table S1). A phylogenetic analysis revealed that the bacterial diversity in the larval digestive tract comprised four main phyla, namely, *Pseudomonadota*, *Actinobacteria*, *Bacillota*, and *Bacteroidota*, with their relative abundance varying depending on the diet (SD or FD). Pseudomonadota representatives dominated in both sample types, while *Bacillota* constituted the least abundant group. The larvae reared on the SD were characterized by the presence of *Micrococcus* and *Brucella* genera within the classes *Actinomycetia* (phylum *Actinomycetota*) and *Alphaproteobacteria* (phylum *Bacteroidota*), whereas the FD-fed larvae predominantly contained *Citrobacter* and *Pseudomonas* within the class *Gammaproteobacteria* (phylum *Pseudomonadota*). Both larval groups shared common bacterial genera, including *Klebsiella*, *Enterobacter*, *Citrobacter*, and *Bacillus*. Diet-specific bacterial taxa were identified: SD-associated *Micrococcus*, *Curtobacterium*, *Brucella*, and *Sphingobacterium* versus FD-associated *Raoultella*, *Serratia*, *Kluyvera*, *Pseudomonas*, and *Glutamicibacter* (Figure 2 and Figure 3).

## 4. Discussion

The gut microbiota of saprophagous insects plays a crucial role in nutrient cycling and host physiology. This is well-supported by numerous studies demonstrating the involvement of intestinal microbes in digestion, nutrient acquisition, detoxification, and immune modulation across various insect groups, including termites [16,54], beetles [55], and lepidopterans [56,57,58].

While culture-independent methods provide valuable insights into the overall microbial community structure, culture-dependent approaches allow for the isolation and characterization of specific bacterial strains, enabling the investigation of their functional properties and potential applications. It is important to acknowledge that culture-based methods only capture a fraction of the total microbial diversity present in the gut. This study focused on isolating and characterizing culturable microbes from the gut of *Z. atratus* larvae, recognizing that these represent only a portion of the overall intestinal community.

This study employed culture-based methods to isolate microbes from the intestinal tract of *Z. atratus* larvae reared on an SD and an FD, representing different trophic specializations. The FD was formulated to mimic the natural feeding preferences of tenebrionid beetles and their saproxylic larvae under wild conditions, while the SD represented a conventional rearing diet typically used for mass-producing larvae as amphibian and reptilian feed in insectariums and zoological facilities. Our findings revealed both conserved patterns of culturable microbiota in saprophagous insects and specific diet-dependent modifications, consistent with the current understanding of the plasticity of insect microbial communities [59,60,61,62].

The identified physiological groups of microorganisms (amino acid autotrophs, enterobacteria, yeasts, mold fungi, and cellulolytic bacteria) are characteristic of many detritivores species [63]. Of particular note is the dominance of enterobacteria in both dietary groups (74.33 × 10^3^ CFU/g in SD and 68.91 × 10^3^ CFU/g in FD), confirming their crucial role in nitrogen metabolism and vitamin synthesis [38]. The observed differences in the abundance of amino acid autotrophs (441.33 × 10^3^ CFU/g in the SD versus 298.45 × 10^3^ CFU/g in the FD) likely reflect adaptations to different protein sources, a phenomenon previously documented in other coleoptera species [64]. Further investigation of these isolates could reveal specific mechanisms of amino acid metabolism.

The most striking difference was observed in the fungal community composition. The significant increase in the abundance of yeasts under the FD (58.72 × 10^3^ CFU/g) compared to the SD (12.15 × 10^3^ CFU/g) supports their presumed role in fungal cell wall degradation [28]. The low abundance of mold fungi (2.67 × 10^3^ CFU/g) may be attributed to both bacterial competitive inhibition [65] and the specific methodological constraints of our isolation approach.

The predominance of aerobes and facultative anaerobes in *Z. atratus* distinguishes it from many xylophagous insects with anaerobic symbionts [54]. This likely relates to its simple intestinal structure lacking specialized fermentation chambers [38,64] and the high aeration of its substrate. These features prompted our focus on investigating the composition and taxonomic structure of aerobic culturable bacteria inhabiting the intestinal tract of *Z. atratus* larvae. Our study revealed that the gut bacterial communities of *Z. atratus* larvae are strongly influenced by diet, with distinct taxonomic profiles emerging between larvae reared on the SD and FD. While the overall phylum-level diversity (based on culturable isolates) was similar between groups—with *Pseudomonadota* dominating in both cases—significant differences were observed at finer taxonomic resolutions. This pattern aligns with findings in other *Coleoptera* species, where diet-induced shifts primarily occur at the genus level, while the phylum-level structure remains stable [63]. The prevalence of *Pseudomonadota* is consistent with that in previous reports in various saproxylic beetles, suggesting that this phylum may represent a core component of the tenebrionid gut microbiome, playing a fundamental role in nutrient metabolism [55,66,67,68,69,70]. Further studies are needed to determine whether these cultured isolates are representative of the overall *Pseudomonadota* diversity in the *Z. atratus* gut.

The influence of diet was particularly evident in the differential enrichment of specific bacterial genera among the culturable isolates. The larvae fed the SD showed a higher abundance of *Micrococcus* and *Brucella* isolates, whereas those reared on the FD exhibited a predominance of *Citrobacter* and *Pseudomonas* isolates. These differences likely reflect functional adaptation to distinct dietary substrates. While these culture-based observations do not capture the entire microbial community, they do suggest that the fungal-based diet potentially selects for culturable microbes capable of interacting with fungal cell wall components [55,71]. Notably, several genera (*Klebsiella*, *Enterobacter*, *Citrobacter*, and *Bacillus*) were represented in cultures from both diets, suggesting that they may contribute essential functions, perhaps independent of the specific dietary input [38,63]. This observation supports the concept of coexistence in the insect gut of both diet-acquired and host-selected microbiota components [38,72,73]; however, it is important to note this is based on a culture analysis and may not represent the whole picture

The presence of diet-specific bacterial taxa among the culturable isolates may be explained by two non-exclusive mechanisms: (1) direct acquisition from the food substrate and (2) ecological filtering by the gut environment [63,74]. Several identified genera—including some that discriminated between diets—have been previously isolated from fungal cultures and decaying wood [71], supporting the substrate acquisition hypothesis. This aligns with studies demonstrating that insect gut microbiota frequently incorporates transient microbes derived from food and environmental sources [63]. However, the persistence of core taxa across different diet types suggests that these culturable microbes may have established stable symbiotic relationships with the host. Such resident bacteria likely contribute to essential physiological functions, including nutrient supplementation and pathogen defense [14]. This dual pattern reflects the dynamic nature of insect–microbe interactions, where environmentally acquired microorganisms coexist with host-selected symbionts.

Our findings align with observations in other wood-associated insects where diet shapes the microbial community structure [75]. For instance, fungus-feeding bark beetles and termites frequently harbor *Pseudomonas* and *Enterobacter* species capable of degrading plant and fungal polysaccharides [15,76]. The presence of similar taxa in the FD-fed *Z. atratus* larvae suggests convergent microbial adaptation to fungivorous diets. However, unlike termites that utilize anaerobic symbionts for lignocellulose digestion [54], *Z. atratus* relies on aerobic and facultatively anaerobic bacteria, which aligns with our focus on culturing aerobic and facultatively anaerobic bacteria.

Several methodological considerations should be noted. While our culture-dependent approach provides a valuable isolation of viable strains, it inevitably underestimates the total microbial diversity by excluding unculturable taxa and strict anaerobes [77]. The culture-dependent approach limits our knowledge of microbial diversity. Recent culture-independent studies have revealed significantly richer gut communities in various insects [78,79,80], suggesting that our data represent only a portion of the whole microbiome. Furthermore, genus-level identification limits functional predictions, as metabolic capabilities can vary substantially among closely related strains [81]. Some researchers have demonstrated that not every microbial taxon identified in insects necessarily serves a specific function [60].

To gain deeper insights into the functional potential of these microbial communities and their interactions with the host, future studies employing metagenomic sequencing and metatranscriptomics will be essential [16]. While our study provides a phenotypic characterization of diet-induced changes in culturable microbes from the gut of *Z. atratus*, a more comprehensive functional analysis would significantly enhance our mechanistic understanding of these microbial adaptations. For instance, the observed enrichment of Citrobacter and Pseudomonas in the FD-fed larvae aligns with their established roles in lignocellulose degradation and phenolic compound detoxification in other insect systems [55,75]. However, the precise metabolic pathways enabling these genera to thrive on fungal wood substrates remain unclear. Future studies of enzyme function can help determine this.

The obtained data advance our understanding of saprophagous insect nutritional ecology. These findings offer potential perspectives for manipulating select bacterial isolates in applied contexts [82]. The ecological implications of diet-induced microbiome changes warrant further investigation. Such investigations would further clarify the ecological significance of diet–microbiota interactions in natural environments, particularly for nutrient cycling in decaying wood ecosystems. While our current methodology focused on culturable aerobic bacteria, future work will integrate anaerobic cultivation with omics approaches to capture the full functional potential of the gut microbiome. These efforts will bridge the gap between taxonomic composition and functional ecology, advancing applications in waste bioconversion and sustainable insect farming.

## 5. Conclusions

Our study demonstrates that dietary modifications in the darkling beetle *Z. atratus* significantly alter the structure of its culturable intestinal microbial communities. For the first time, we provide data on the composition of aerobic culturable bacteria and fungi isolated from larvae reared on wood-based substrates. The results indicate that different diets lead to distinct culturable gut microbiota profiles. While we acknowledge that these culturable isolates represent only a fraction of the overall gut community, they suggest potential impacts on the larvae’s digestive capabilities. The broader taxonomic structure of the isolated bacterial community remains relatively stable; however, dietary changes cause significant shifts in specific bacterial lineages. These findings enhance our understanding of how specific culturable gut microorganisms contribute to host adaptation to environmental conditions. This study opens new research avenues for investigating key processes such as lignin, cellulose, and hemicellulose biodegradation; nitrogen fixation; and the detoxification of wood components. Specifically, the observed shifts in the culturable gut microbiota composition associated with fungal-based diets suggest potential applications in enhancing the bioconversion of lignocellulosic waste materials (specifically through the use of isolates exhibiting such activities). By targeting the microbiota of *Z. atratus* larvae, it may be possible to enhance their ability to degrade complex plant matter and convert it into valuable biomass or other useful products. Furthermore, characterizing specific culturable isolates in *Z. atratus* digestion and nutrient acquisition could inform strategies for optimizing insect rearing practices, improving feed efficiency, and enhancing overall larval growth and health. The results establish a foundation for future research on functional microbiome manipulation in economically important insect species, potentially enabling the optimization of their cultivation and biotechnological applications.

## Figures and Tables

**Table 1:** Components of experimental diets for the rearing of *Zophobas atratus* under laboratory conditions. Composition according to Kuprin et al. [41], with modifications.

Name of Component	Fungal-Based Diet, %	Standard Diet, %	Control Diet, %
Sawdust of *Ulmus japonica*	24.0	-	90
Distilled water	62.9	10	10
Wheat flakes and bran	-	90	-
Mycelium of *Pleurotus citrinopileatus*	5.0	-	-
Feed yeast	2.0	-	-
Ascorbic acid	0.9	-	-
Sucrose	4.0	-	-
Agar	1.2	-	-

## Data Availability

The datasets presented in this study can be found in GenBank under numbers PP905376-PP905385 and PP906184-PP90620. Data are available upon request to the corresponding author of this MS.

## Supplementary Materials

The following supporting information can be downloaded at: https://www.mdpi.com/article/10.3390/biology14070824/s1, Table S1: Taxonomic classification of the obtained bacterial isolates.
