# Supplementary material for: Isolation and Characterization of Cultivable Microbes from the Gut of Zophobas atratus (Coleoptera: Tenebrionidae) Larvae Reared on Two Types of Artificial Diets
Source: Biology (Basel). 2025 Jul 7;14(7):824. doi: 10.3390/biology14070824 (PMC12292095; doi:10.3390/biology14070824)
Supplement: Supplementary file 1 [file biology-14-00824-s001.zip › biology-3670531-supplementary.pdf]

Table S1. The taxonomic classification of the obtained bacterial isolates.

№	Strain designation	Isolation source	GenBank accession number	Species	Genus	Family	Class	Phylum
1	SD-14/21	Larval intestinal tract (standard diet, SD)	PP905376	*Klebsiella sp.*	*Klebsiella*	*Enterobacteriaceae*	*Gammaproteobacteria*	*Pseudomonadota*
2	SD-4/21		PP905377	*Enterobacter sp.*	*Enterobacter*			
3	SD-18/21		PP905378	*Citrobacter sp.*	*Citrobacter*			
4	SD-1/21		PP905379	*Micrococcus sp.*	*Micrococcus*	*Micrococcaceae*	*Actinomycetia*	*Actinomycetota*
5	SD-21/21		PP905380	*Micrococcus sp.*				
6	SD-12/21		PP905381	*Curtobacterium sp.*	*Curtobacterium*	*Microbacteriaceae*		
7	SD-8/21		PP905382	*Brucella sp.*	*Brucella*	*Brucellaceae*	*Alphaproteobacteria*	*Bacteroidota*
8	SD-10/21		PP905383	*Brucella sp.*				
9	SD-15/21		PP905384	*Sphingobacterium sp.*	*Sphingobacterium*	*Sphingobacteriaceae*	*Sphingobacteriia*	
10	SD-20/21		PP905385	*Bacillus sp.*	*Bacillus*	*Bacillaceae*	*Bacilli*	*Bacillota*
11	FD-18/23	Larval intestinal tract (fungal-based diet, FD)	PP906184	*Klebsiella sp.*	*Klebsiella*	*Enterobacteriaceae*	*Gammaproteobacteria*	*Pseudomonadota*
12	FD-20/23		PP906185	*Klebsiella sp.*				
13	FD-3/23		PP906186	*Enterobacter sp.*	*Enterobacter*			
14	FD-22/23		PP906187	*Enterobacter sp.*				
15	FD-1/23		PP906189	*Citrobacter sp.*	*Citrobacter*			
16	FD-4/23		PP906190	*Citrobacter sp.*				
17	FD-6/23		PP906191	*Citrobacter sp.*				
18	FD-17/23		PP906192	*Citrobacter sp.*				
19	FD-23/23		PP906193	*Citrobacter sp.*				
20	FD-35/23		PP906194	*Citrobacter sp.*				
21	FD-32/23		PP906195	*Raoultella sp.*	*Raoultella*			
22	FD-33/23		PP906196	*Raoultella sp.*				
23	FD-2/23		PP906188	*Serratia sp.*	*Serratia*			
24	FD-10/23		PP906197	*Kluyvera sp.*	*Kluyvera*			
25	FD-19/23		PP906198	*Pseudomonas sp.*	*Pseudomonas*	*Pseudomonadaceae*		
26	FD-34/23		PP906199	*Pseudomonas sp.*				
27	FD-37/23		PP906200	*Pseudomonas sp.*				
28	FD-9/23		PP906201	*Glutamicibacter sp.*	*Glutamicibacter*	*Micrococcaceae*	*Actinomycetia*	*Actinomycetota*
29	FD-25/23		PP906202	*Bacillus sp.*	*Bacillus*	*Bacillaceae*	*Bacilli*	*Bacillota*
